# Recent Trends in anti-tumor mechanisms and molecular targets of celastrol

**DOI:** 10.7150/ijbs.99592

**Published:** 2024-10-07

**Authors:** Yongping Zhu, Yuqing Meng, Junzhe Zhang, Rui Liu, Shengnan Shen, Liwei Gu, Yin-kwan Wong, Ang Ma, Xin Chai, Ying Zhang, Yanqing Liu, Jigang Wang

**Affiliations:** 1 State Key Laboratory for Quality Ensurance and Sustainable Use of Dao-di Herbs, Artemisinin Research Center, and Institute of Chinese Materia Medica, China Academy of Chinese Medical Sciences, Beijing 100700, China.; 2 College of Animal Science and Technology, Henan Agricultural University, Zhengzhou 450002, China.; 3 Department of Physiology, Yong Loo Lin School of Medicine, National University of Singapore, Singapore 117600, Singapore.; 4 Department of Critical Care Medicine, Guangdong Provincial Clinical Research Center for Geriatrics, Shenzhen Clinical Research Center for Geriatric, Shenzhen People's Hospital (The Second Clinical Medical College, Jinan University; The First Affiliated Hospital, Southern University of Science and Technology), Shenzhen 518020, China.; 5 State Key Laboratory of Antiviral Drugs, School of Pharmacy, Henan University, Kaifeng, China.

**Keywords:** celastrol, pharmacology, mechanisms, targets

## Abstract

Celastrol, a compound derived from traditional Chinese medicine, has therapeutic effects and has been used to treat inflammation-related diseases, cancer, cardiovascular diseases, and neurodegenerative diseases. However, current reviews lack a comprehensive and systematic summary of the anti-tumor mechanisms and molecular targets of celastrol. For this reason, this paper reviews the anticancer properties of celastrol and the molecular mechanisms underlying its anticancer effects. This paper primarily focuses on the mechanism of action of celastrol in terms of inhibition of cell proliferation and regulation of the cell cycle, regulation of apoptosis and autophagy, inhibition of cell invasion and metastasis, anti-inflammation, regulation of immunotherapy, and angiogenesis. More importantly, the target proteins of celastrol identified by chemical proteomics or other methods are highlighted, providing detailed targets with novel therapeutic potential for anti-tumor treatment. In addition, we describe the side effects and strategies to improve the bioavailability of celastrol. In summary, this paper analyzes celastrol, a natural compound with therapeutic effects and clear targets, aiming to draw more attention from the scientific and pharmacological communities and accelerating its clinical application for the benefit of cancer patients.

## 1. Introduction

Cancer remains one of the most lethal human diseases, with high annual incidence and mortality rates. The latest cancer statistics report indicates 19.3 million new cases and 10 million deaths worldwide in 2020 alone 1. Surgery, chemotherapy, and radiotherapy are the preferred, recognized, and widely used cancer treatments globally 2-4, with immunotherapy also playing an increasingly important role in tumor treatment in recent years 5,6. Despite the availability of therapeutic modalities and targeted drugs, cancer continues to be the leading cause of death worldwide. Therefore, further research into new strategies and the discovery of potential new drugs for cancer treatment is urgently needed.

Natural resources have become a significant part of the drug market, overshadowing synthetic compounds in drug development over the past half-century. Recently, natural medicinal plants and bioactive molecules have attracted immense interest for their potent efficacy, minimal side effects, lower cost, and in some cases, extraordinary anti-inflammatory, anti-obesity, anticancer, and neuroprotective properties 7. It is widely believed that such substances can be used as a reasonable alternative to synthetic substances. As a prominent natural product molecule, celastrol has been identified as one of the five most promising natural pharmaceutical products. Also known as tripterygium, celastrol is a red acicular crystal and a quinone methyl pentacyclic triterpenoid compound isolated from *Tripterygium wilfordii*, a traditional Chinese medicine 8. Recent studies have highlighted the diverse pharmacological activities of celastrol, including anti-inflammatory (9), anti-diabetes and obesity 10-12, immunomodulatory 13, and particularly, anti-tumor effects 14. Celastrol displays broad-spectrum and efficient anti-tumor activity. It has been reported to inhibit various cancer cells, including lung, ovarian, stomach, liver, leukemia, colon, and breast cancers 15, and to enhance the sensitization effects of radiotherapy and chemotherapy 16.

This review comprehensively and systematically summarizes the anti-tumor effects of celastrol, including its molecular mechanisms, target proteins, drug combinations, bioavailability, and potential side effects. In particular, the mechanism of action and targets of celastrol are highlighted. Drugs generally prevent and treat diseases by acting on these drug targets and further modulating up- and downstream signaling pathways. Identifying drug targets is therefore essential for research and development of novel cancer drugs and therapies 17. We hope that this review will provide novel and comprehensive design ideas and a theoretical basis for further research on celastrol, as well as theoretical guidance for its clinical prescription and acceleration of clinical trials.

## 2. Anti-cancer potential and molecular mechanisms of celastrol

### 2.1 Celastrol inhibits cell proliferation and regulates the cell cycle

The uncontrolled proliferation of tumor cells results from dysregulation of the cell cycle, and targeting this dysregulation is a key strategy in current cancer therapies. Studies have demonstrated that celastrol affects the cell cycle, including the modulation of cyclins and cyclin kinases, and inhibits the proliferation of tumor cells across various cancer types. Li *et al.* reported that treating ovarian cancer cells with celastrol led to the inhibition of Pin1 protein expression, resulting in the down-regulation of Cyclin D1, CDK2, and CDK4, and the arrest of the cell cycle in the G2/M phase 18. Similarly, other studies have shown that celastrol upregulates Cyclin B1 and downregulates Cyclin E in a dose-dependent manner, effectively blocking the cell cycle in the G2/M phase 19. Celastrol also enhances p21 and p27 expression while decreasing Cyclin D1 and Cyclin E levels, inhibiting cell proliferation and leading to G1 phase arrest in multiple myeloma cells 20. Additionally, celastrol significantly increases the amount of reactive oxygen species (ROS) in colon cancer cells at both the cytoplasmic and mitochondrial levels, causing cell cycle arrest in the S phase 21. Treatment of a human osteosarcoma cell line with celastrol resulted in cell cycle arrest at the G2/M phase, followed by apoptosis through the activation of the OS/JNK signaling pathway 22. In gastric cancer cells, celastrol induced G2/M cell-cycle arrest by inhibiting the miR-21-mTOR signaling pathway and increasing the p27 protein level 23. Furthermore, celastrol caused cell cycle arrest in the G2/M phase of nasopharyngeal carcinoma by upregulating the mitogen-activated protein kinase (p38 MAPK) and extracellular signal-regulated Kinase (ERK) pathways, triggering apoptosis via both endogenous and exogenous pathways, thus exerting an anti-tumor effect 24. In essence, celastrol impedes the growth and proliferation of cancer cells by influencing cell cycle progression.

### 2.2 Celastrol regulates cell apoptosis

Apoptosis, a process of programmed cell death under physiological or pathological conditions, is influenced by the regulation of intracellular genes and some extracellular factors. It affects various processes including growth, metabolism, immunological defense, cell differentiation, nervous system damage, and tumor development 25. The apoptosis of tumor cells is closely associated with abnormal expression of the endogenous apoptosis pathway and the exogenous pathway 26. Studies have demonstrated that celastrol can induce apoptosis in various tumor cells through multiple mechanisms, such as the activation of caspases 27-30. Celastrol inhibits tumor cell proliferation, reduces migration and invasion capabilities, and induces cell cycle arrest by increasing the expression of cleaved-PARP, cleaved-caspase-3, cleaved-caspase-8, and cleaved-caspase-9, ultimately leading to apoptosis 27-28.

The FAS gene belongs to the TNF receptor family. After the Fas protein binds with its ligand (Fas-L), target cells can be induced to undergo apoptosis via activating the caspase cascade. Abnormal expression of Fas and FasL has been shown to inhibit apoptosis in normal cells and promote the survival of poorly differentiated cells, leading to tumor formation 31. The administration of celastrol has been reported to induce apoptosis by activating mitochondria and Fas/Fas related pathways in non-small cell lung cancer cells 32. In glioma cells, celastrol activates the cell death receptor pathway by increasing the expression levels of death receptor 5 (DR5), caspase-8, caspase-3, and PARP proteins, thereby inducing apoptosis 33. Additionally, celastrol increases the expression of TNF receptor superfamily members (TNRSF) 1A and 10B, and TNFRSF1A is associated with the death domain, Fas, and Fas-associated via the death domain, leading to cell cycle arrest in the G1 phase and G2/M phase, and inducing apoptosis 34.

Celastrol promotes apoptosis by inducing the mitochondrial apoptosis pathway 35-36. Mitochondria, the direct energy suppliers of cellular activities, undergo structural remodeling and increased outer membrane permeability during apoptosis, leading to the release of cytochrome c (CytC) which activates downstream caspases involved in the apoptosis process 37. Celastrol induces apoptosis in acute promyelocytic leukemia (HL-60 and NB-4) by inhibiting cysteine metabolism, increasing ROS levels, and activating the mitochondrial apoptosis pathway 35. Furthermore, celastrol triggers the mitochondrial apoptosis pathway by upregulating the expression of CytC and the pro-apoptotic protein Bax, activating caspase-3 and caspase-9, and leading to the cleavage of PARP in human osteosarcoma cells 36. Studies have also shown that celastrol directly binds to peroxiredoxin-2 (Prdx2) and inhibits its enzyme activity, increasing ROS levels and resulting in ROS-dependent endoplasmic reticulum (ER) stress, mitochondrial dysfunction, and apoptosis in gastric cancer cells 38.

Celastrol also induces apoptosis through the ER stress (ERS) emergency pathway 39-41. The ER, a major cellular organelle that modifies and secretes proteins, activates ERS under constant and intense stimulation, leading to protein folding errors and excessive accumulation of unfolded proteins, triggering the unfolded protein response and initiating apoptosis. ERS regulates tumor cell proliferation, migration, and apoptosis 42. Chen and colleagues revealed that celastrol inhibits the expression levels of ERS-related proteins (Bip, PERK, p-PERK, Ero1-Lα and calnexin), mitochondrial apoptosis-related proteins, and autophagy-related proteins in human osteosarcoma cells, inducing apoptosis 39. Similarly, celastrol upregulates the expression of CHOP, Bip, XBP1s, and IRE1 proteins, increased the transcription of ERS target genes such as BIM, induced Bax translocation into mitochondria, and activated the ER and mitochondrial apoptosis pathways in cervical cancer HeLa cells 40. Moreover, in non-small cell lung cancer (NSCLC), celastrol significantly increases intracellular ROS levels and triggered the activation of the ERS pathway, ultimately leading to apoptosis 41. Although celastrol induces apoptosis in different ways, these pathways are not independent of each other but are closely linked. For example, both the death receptor pathway and the mitochondrial pathway can activate caspase proteins, which in turn leads to apoptosis. Celastrol also does not necessarily induce apoptosis through only a single signaling pathway. For example, it can induce apoptosis via both the ER pathway and the mitochondrial pathway 39-40. In conclusion, celastrol exerts its anti-tumor effects partially through the induction of apoptosis by affecting one or more apoptotic pathways. However, how celastrol regulates multiple apoptotic pathways and the links between these pathways still need to be further studied.

### Celastrol regulates cell autophagy

Autophagy is a form of programmed cell death in which cellular material is transported into the lysosome for degradation, allowing for the turnover of cellular components, and providing energy and macromolecular precursors 43. In the context of cancer, autophagy serves a dual role, both by inhibiting tumorigenesis and by supporting tumor progression. It is generally accepted that autophagy suppresses tumor formation, yet evidence indicates that established tumors rely on the autophagic process for sustained cell growth and increased metabolic activity, thus depending on autophagy for maintenance 44. Previous studies have demonstrated that celastrol can induce autophagy in cancer cells 45. Treatment with celastrol has been shown to increase the expression of Atg5 and Atg7, facilitate the formation of autophagosomes, and promote the degradation of p62, thereby enhancing autophagy activity and reducing cell viability 46. In human colorectal cancer cells, celastrol triggers apoptosis and autophagy by inhibiting Nur79, leading to increased expression of Atg7 and activation of the Nur77/Atg7 signaling pathway 47. Furthermore, celastrol activates the LXRα signaling pathway, inducing autophagy and lipid droplet degradation, promoting ABCA1-mediated cholesterol efflux and impairing EMT progression, thereby playing an anti-invasive and anti-metastastic role in clear cell renal cell carcinoma 48. Studies have also shown that celastrol upregulates the conversion of autophagy markers such as microtubule-associated protein 1 light chain 3I (LC3I) to LC3II, thereby inducing autophagy in cancer cell lines A549, PC-3, and HeLa 49. Additionally, celastrol-treated gastric cancer cells exhibited enhanced levels of autophagy-related proteins Atg5, Atg7, Beclin1, and LC3I and II, which induce autophagy 50. All these data indicate that celastrol can induce autophagy in cancer cells which inhibits tumorigenesis and suppresses tumor progression, and more specific molecular mechanisms and *in vivo* validation of the regulation of autophagy by celastrol are worthy of further study.

### 2.4 Celastrol inhibits cell invasion and metastasis

Metastasis occurs when tumor cells invade lymphatic vessels, blood vessels, or body cavities from their original site, and are transported via the bloodstream or lymph flow to another site or organ to continue growing, forming tumors identical to the primary tumor. Data indicate that over 90% of cancer-related deaths are caused by metastasis 51. Chemokines, small cytokines or signaling proteins secreted by cells, constitute a family of more than 60 members with molecular weights ranging from 8 to 10 kDa 52. Chemokine receptors (CXCRs) are G-protein-linked transmembrane proteins that interact with these cytokines to facilitate the spread of malignant cells, making drugs capable of blocking this interaction potential inhibitors of tumor cell metastasis 53. CXCR4 is a crucial receptor involved in the interaction between tumor cells and their microenvironment. Studies have shown that celastrol decreases CXCR4 expression in a dose-dependent manner and downregulates related downstream pathways, including the PI3K/AKT pathway, significantly reducing tumor cell proliferation and migration capacity 54-55. Additionally, celastrol indirectly influences the expression of miR-223-3p by downregulating circ_SLIT3 and affecting CXCR4, thereby restraining the migration and invasion of HCC cells 56.

Mounting evidence suggests that extracellular proteases, such as matrix metalloproteinases (MMPs), which are capable of degrading various components of the extracellular matrix (ECM), play crucial roles in tumor invasion and metastasis by breaking down the histological barriers to tumor cell invasion 57. Celastrol effectively inhibits cell migration and invasion in human osteosarcoma cells through down-regulating MMP2 and MMP9, by inhibiting the PI3K/AKT/NF-κB signaling pathway 58-60. Furthermore, celastrol suppresses the expression of MMP3 and MMP7 in colorectal cancer cells by inhibiting the PI3K/AKT pathway, thereby affecting their proliferation and migration of 61. Additionally, further evidence suggests that celastrol downregulates the phosphorylation of focal adhesion kinase (FAK) by activating the p38 MAPK signaling pathway, blocking the adhesion of the mouse melanoma cell line B16F10 and human lung cancer cell line 95-D to the extracellular matrix, and thereby inhibiting adhesion plaque-dependent cell migration and invasion 62.

The tumor microenvironment plays a vital role in tumor cell migration, invasion and angiogenesis 63. It has been found that celastrol can reverse macrophage polarization from M2 to M1 *in vivo* and *in vitro* by affecting the colorectal tumor microenvironment, as well as mechanistically polarizing macrophages through the MAPK signaling pathway 64. In addition, celastrol and siRNA have been delivered to retinoblastoma cells in the form of polymeric micelles to achieve synergistic anti-tumor and anti-angiogenic effects. The co-delivery system specifically and synergistically inhibited the expression of HIF-1α and VEGF in retinoblastoma cells, inhibited the HIF-1α /VEGF/VEGFR signaling pathway, ameliorated the hypoxic microenvironment of the tumors, and hindered the proliferation, migration, and invasion of vascular endothelial cells 65. In summary, celastrol inhibits cell invasion and metastasis by influencing the tumor microenvironment and the expression of cytokines.

### 2.5 Celastrol regulates tumor immunotherapy

Immunotherapy has emerged as a powerful clinical strategy for the treatment of cancer. Advanced biomaterials and delivery systems, such as those utilizing T-cell delivery or nanoparticles, can be used to effectively utilize immunotherapies and increase their efficacy while reducing toxic side effects. Studies have shown that celastrol in combination with nanoparticle delivery systems has promising applications in melanoma immunotherapy 66-68. It was found that celastrol not only induced immunogenic cell death (ICD), but also down-regulated PD-L1 expression in tumor cells, activated both dendritic cells and T cells, and interrupted the PD-1/PD-L1 pathway between T cells and tumor cells. In a bilateral tumor model, intratumoral injection of a high concentration of celastrol nanoemulsion effectively activated the immune system, thereby suppressing both treated and distant untreated tumors (i.e., the ascites effect) in mice 66. Wang *et al.* constructed an ER-targeted celastrol nanoparticle, TSE-CEL/NP, which specificlly aggregated in the ER and induced ER stress. Excessive ER stress exposed the cells to calcreticulin, which released ATP and HMGB1 extracellularly. This enhanced the recruitment and maturation of dendritic cells, which in turn increased the proliferation of cytotoxic T lymphocytes (CD8+ T cells), thereby enhancing the effectiveness of immunotherapy against melanoma 67. In addition, celastrol directly binds to IL-2, disrupts the binding of IL-2 and CD25, markedly inhibits the proliferation and signaling of IL-2-dependent mouse T cells, and increases the number of CD8+ T cells, thereby inhibiting tumor growth. The group also reported that celastrol failed to inhibit tumor growth in T cell-deficient BALB/c nude mice, suggesting that its activity was mediated by T cell responses. In addition, combination therapy with low-dose celastrol and a TNFR2 antagonist synergistically improved the efficacy of the single agent by increasing the ratio of intratumoral CD8+ T cells/Treg cells and inhibiting the expression of Foxp3. Celastrol thus exerts antitumor activity by targeting IL-2 to inhibit CD25 binding and mediating T cell responses, and has the potential to be a candidate for melanoma combination therapy in cancer immunotherapy 68. In conclusion, while celastrol has shown great potential in tumor immunotherapy, few studies have been conducted in this area and more research is needed to elucidate the role of celastrol in immunotherapy.

### 2.6 Anti-inflammatory properties of celastrol

The immune system plays crucial roles in both tumor development and treatment. Adaptive immunity works to prevent or suppress tumors through immune surveillance, whereas innate immunity and inflammation often promote the onset and malignant progression of new cancers 69. Chronic inflammation induced by certain infectious diseases has been reliably linked to cancer development. Thus, anti-infective agents play a significant role in reducing the burden of inflammation-related cancers. Nuclear factor-κB (NF-κB) and signal transducer and activator of transcription 3 (STAT3) are key regulators of inflammation, cell transformation, tumor cell survival, proliferation, and metastasis 70. NF-κB is an important nuclear transcription factor involved in multiple complex biological processes, including immune regulation, inflammation, cell proliferation, and apoptosis, and is often overexpressed in various tumors, such as myeloma, breast, ovarian, and prostate cancers 71-72. As an NF-κB inhibitor, celastrol suppresses the activity and expression of NF-κB and its associated genes, thereby inhibiting the proliferation, migration, and invasion of tumor cells. In prostate adenocarcinoma cells, celastrol modulated the protein expression of IκBα, IKKα (kappa B inhibitory kinase α subunit), p50, and NF-κB, promoted the degradation of the anti-apoptotic protein Mcl-1, and activated the pro-apoptotic protein Noxa. It also blocked the expression of IL-6, ultimately inhibiting tumor cell proliferation and invasion 73-74. Celastrol additionally obstructed NF-κB and its downstream gene products, such as CXCR4 and MMP9, and reduced serum IL-6 and TNF-α levels to inhibit cell invasion and migration *in vivo* 75. Studies have indicated that celastrol inhibits IκBα phosphorylation, preventing IκBα degradation and NF-κB accumulation. This in turn suppresses the expression and activity of the NF-κB target MMP9, thus blocking the NF-κB pathway and resulting in anti-ovarian cancer cell invasion activity 76. Moreover, celastrol reduces the level of IL-6 by inhibiting the NF-κB signaling pathway in breast cancer cells, inhibiting cell proliferation, migration, and invasion, thereby playing an anti-tumor role 77.

As a nexus of numerous carcinogenic signaling pathways, STAT3 centrally regulates the anti-tumor immune response. It is widely over-activated in cancer cells, playing a crucial role in promoting the expression of immunosuppressive factors and inhibiting the production of key immune activation regulators 78. Research has shown that celastrol inhibits STAT3 by suppressing the activation of upstream kinases c-Src and Janus-activated kinases -1 and -2 (JNK 1/2), impacting tumor cell proliferation 79. By significantly increasing the expression levels of miR-181b and miR-24, celastrol reduces the phosphorylation of STAT3 and the ratio of B-cell lymphoma-2 (Bcl-2)/Bcl-2 associated X (Bax), thereby inhibiting cell proliferation and inducing apoptosis 80. These results provide a theoretical basis for further exploration of the anti-tumor properties of celastrol through the regulation of the immune system.

### 2.7 Celastrol and angiogenesis

During tumor progression, angiogenesis is a complex process often triggered by hypoxia, which leads to increases in angiopoietin, vascular endothelial growth factor (VEGF), and hypoxia-inducing factor (HIF-1) levels. Angiopoietin and VEGF are considered pivotal in tumor angiogenesis 81. The development of new antiangiogenic drugs represents a crucial strategy for treating multiple solid tumors by reducing or eliminating the blood supply to tumor microregions, causing extensive hypoxic necrosis of solid tumor tissues. These drugs are vital in treating certain cancers 82. Previous studies have demonstrated that celastrol inhibits the growth of human glioma xenografts in nude mice by suppressing the expression and transcription of VEGF receptor (VEGFR) 1 and vascular dermal growth factor receptor (VDGFR) 2 83. Another study showed that celastrol inhibited the growth and migration of colorectal cancer cells, and this effect is associated with the inhibited expression of key genes and proteins associated with the angiogenesis pathway including PDGF, MMP-9, Serpin E1 and TIMP-4 84. Additionally, treatment with celastrol has been reported to inhibit tumor growth and angiogenesis *in vitro* and *in vivo* by targeting the mTOR/AKT/S6K kinase signaling pathway 14. Thus, celastrol may partly exert its anti-tumor effects across various cancers by inhibiting angiogenesis. However, further comprehensive studies will be necessary to elucidate the mechanisms behind the anti-angiogenic properties of celastrol.

**Figure 1:**
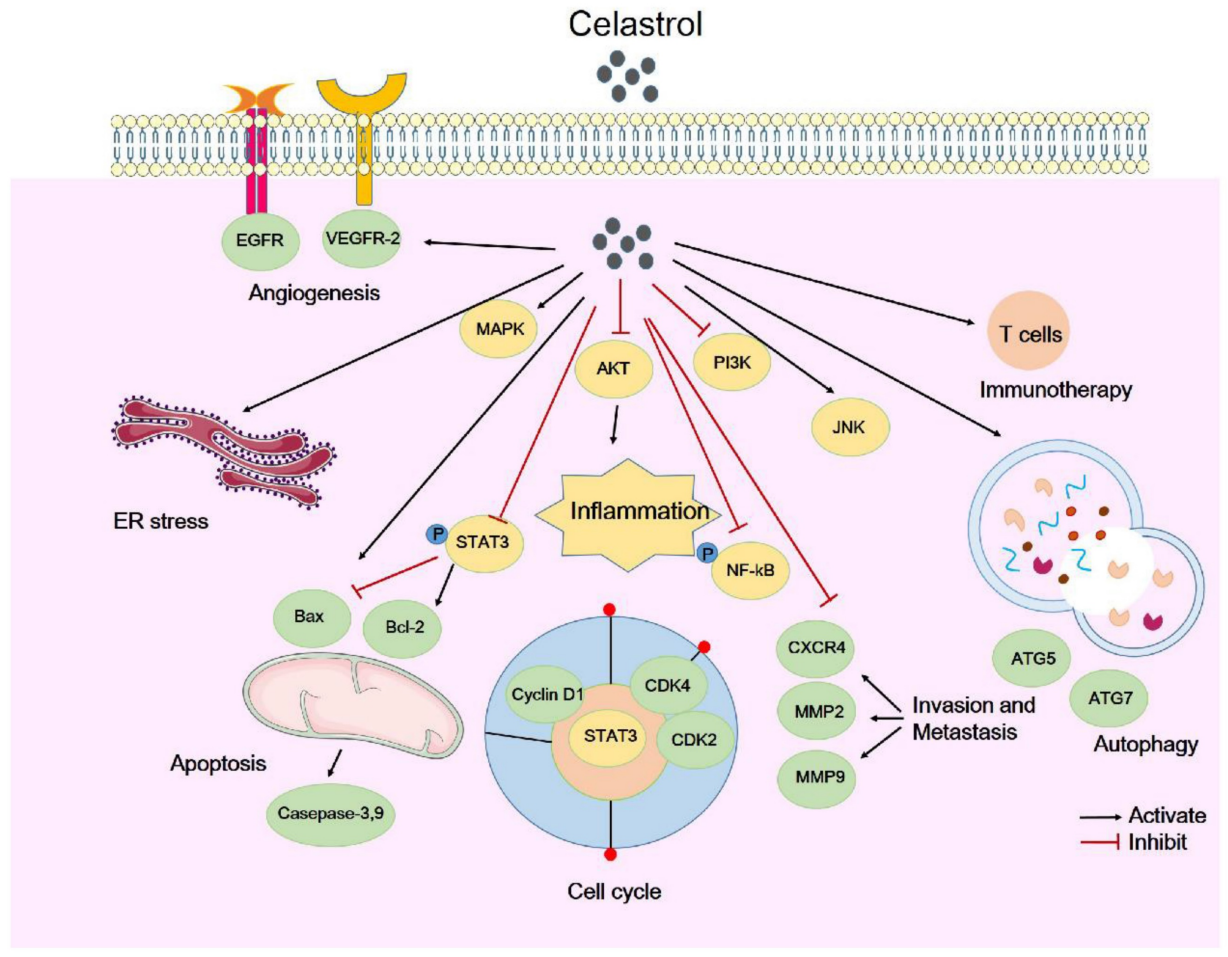
The molecular mechanisms of tumor suppression by celastrol. Celastrol attenuates tumor growth through various mechanisms, including inhibiting cell proliferation and regulating the cell cycle, regulating cell apoptosis and autophagy, inhibiting cell invasion and metastasis, anti-inflammatory properties, regulating tumor immunotherapy and angiogenesis.

In summary, celastrol mainly attenuates tumor growth by inhibiting cell proliferation and regulating the cell cycle, regulating cell apoptosis and autophagy, inhibiting cell invasion and metastasis, and through its anti-inflammatory, antioxidant, and anti-angiogenic activities. Here, we briefly summarize the types of celastrol-induced anticancer activities and their mechanisms (Table 1). The molecular mechanisms by which celastrol inhibits tumors are depicted in Figure 1. Importantly, celastrol exerts these anti-tumor effects by binding to different cellular targets and affecting various upstream and downstream signaling pathways. In the next section, we will focus on these target proteins of celastrol.

## 3. Celastrol and its target proteins

**Figure 2:**
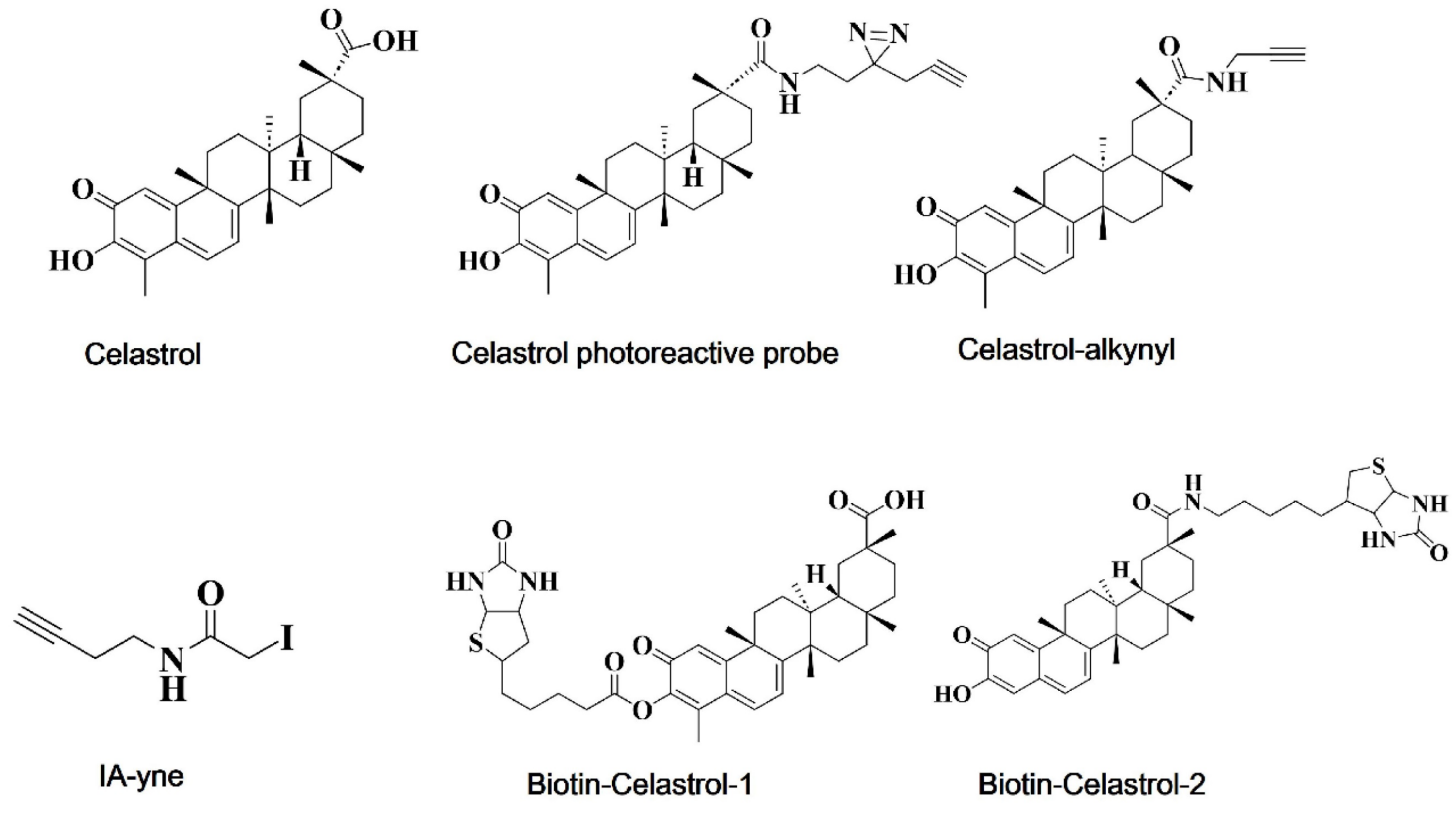
Chemical structures of celastrol and its available probes.

Approximately 123 out of the 154 anticancer drugs approved by the U.S. Food and Drug Administration (FDA) function through protein targets, underscoring the importance of drug target discovery and identification in oncology 85. Drug targets are direct binding sites on biological macromolecules *in vivo*, encompassing proteases, receptors, ion channels, nucleic acids (DNA and RNA), and other biological macromolecules 86-88. The identification of drug targets is crucial for researching and developing novel cancer therapies. Omics technology has become increasingly recognized by scientists for its role in unbiased target prediction and has been widely adopted in the field. Chemical proteomics, a multidisciplinary approach that combines chemical synthesis, mass spectrometry (MS) and cell biology, offers an unbiased platform for identifying small-molecule-protein interactions across a range of natural products and compounds, and has been successfully applied in prominent compounds such as artemisinin, celastrol, curcumin, aspirin, and eupalinolide B 89-93. This summary presents the available probes (Figure 2) and the reported targets of celastrol, along with its mechanism of action (MOA), to enhance the understanding of celastrol targets and its pharmacological actions on cancer 93-96.

**Figure 3:**
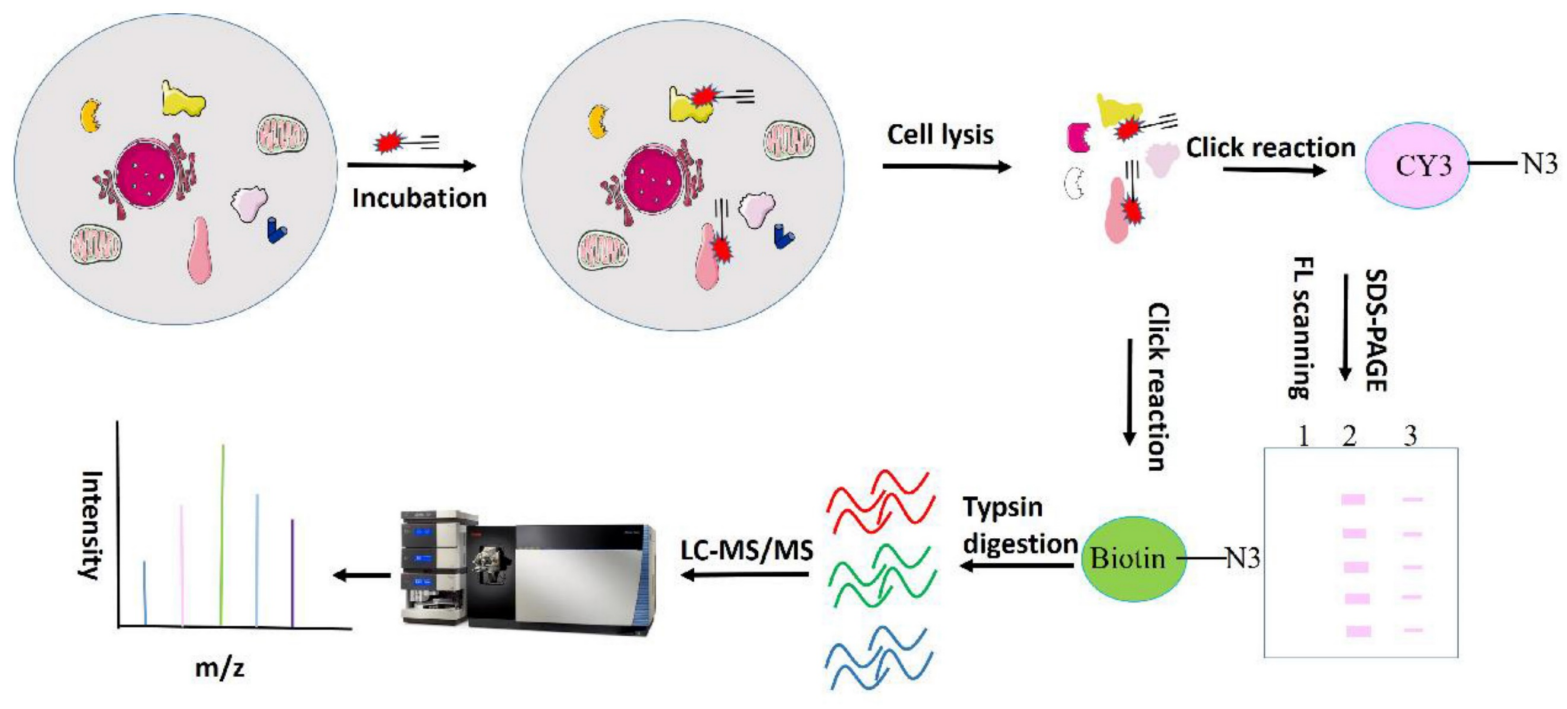
Flow chart of chemical proteomics related to celastrol.

Zhou Y *et al.* have identified over 60 celastrol-binding proteins using iodoacetamide-derived cysteine reaction probes (iodoacetamide-alkyne, IA-yne) via competitive chemical proteomics in human cervical cancer HeLa cells 97. Bioinformatics analysis revealed that celastrol exerts anti-tumor effects by promiscuously interacting with numerous proteins engaged in various cellular pathways and biological processes. Among the identified targets, only three (Hsp90 co-chaperone Cdc37, Annexin A2, and elongation factor 1-alpha 1 (eEF1A)) were consistent with previously-reported celastrol-binding proteins, likely due to celastrol's relatively low protein alkylation efficiency. Another study synthesized a clickable photoreactive probe for comprehensive analysis of targets and identified 700 celastrol-binding proteins, among which celastrol targeted various proteins associated with the biosynthesis, regulation and transport of cholesterol and its metabolites, and is involved in cholesterol metabolism and signaling 95. Klaić and colleagues synthesized and developed an active biotinated celastrol probe and performed affinity pull-down experiments with PANC-1 cell lysates to identify cell targets of celastrol, in which Annexin II and eEF1A were identified. Notably, HSP90 was not determined to be a direct target in their study, but was indirectly targeted by triggering redox imbalances 93. By utilizing an alkyne-linked Cel-p, our research group previously investigated the covalent binding targets and underlying mechanisms of celastrol in liver fibrosis, ischemia stroke, colon cancer and obesity depression comorbidity (79,98-100). We will next discuss the identified celastrol targets and their roles in tumors. A flowchart for chemical proteomics analysis of celastrol is shown in Figure 3.

### 3.1 Celastrol targets HSP90 for anti-tumor effects

Heat shock proteins (HSPs) are considered to be involved in important molecular processes related to cancer development and spread, and are potential clinical targets for cancer treatment and biomarkers for cancer detection. HSP90, a key molecular chaperone, facilitates the folding of various proteins into their mature and stable forms. Given the vital role of HSP90 and its client proteins in tumors, celastrol as a HSP90-targeting compound could represent an important novel regulator of the HSP90 pathway 101. In fact, celastrol treatment is known to lead to the accumulation of ubiquitinated proteins 102, and this accumulation may be due to the inhibition of HSP90 and the stress response, as well as the subsequent redirection of proteins through the proteasome pathway 103. Sreeramulu *et al.* discovered that celastrol covalently binds to Cdc37 and forms disulfide bonds with it, inactivating Cdc37 and disrupting the HSP90-Cdc37 complex 104. Moreover, researchers synthesized a celastrol analog based on its structure and found that the analog significantly disrupted HSP90-Cdc37 protein interaction and induced apoptosis in cancer cells by binding (hydrogen and/or covalent bonding) to Cdc37 105. Owing to their ATP-dependent nature, HSP90 inhibitors typically target ATP-binding pockets, leading to the non-selective degradation of HSP90 client proteins. Celastrol however is known to trigger oligomerization into amyloid fibrils by binding to the cochaperone protein p23, selectively destabilizing steroid receptors over kinase clients 106. Our mass spectrometry research also showed that celastrol directly binds to HSP90 and HSP70, exerting antitumor and neuroprotective effects 99,107. These findings suggest that celastrol may inhibit tumor growth by disrupting the interaction between HSP90 and its client proteins. Although celastrol has been reported to target HSP90 or affect the interaction between HSP90 and its service proteins, more in-depth studies are needed to elucidate how celastrol affects HSP90 and its client proteins, which in turn affect downstream signaling pathways to exert anti-tumor effects.

### 3.2 Celastrol exerts anti-tumor activity by inhibiting STAT3

We previously mentioned that STAT3 plays a central role in modulating anti-tumor immune responses as an intersection of many carcinogenic signaling pathways 78. Through the integration of proteome microarrays, competitive binding, molecular simulation, and surface plasmon resonance (SPR) techniques, STAT3 was identified as a direct binding target of celastrol. Celastrol appears to bind to the coiled-coil domain (CCD, Leu-207) and SH2 domain (Gln-635/Val-637) of STAT3, reducing its tyrosine phosphorylation and nuclear translocation. Interestingly, the overexpression of STAT3 partially counteracted the inhibitory effect of celastrol on STAT3 activity. These findings suggest that celastrol suppresses angiopoietin II-induced hypertrophy and fibrosis in rat primary cardiomyocytes and H9C2 cells by attenuating STAT3 activity 108. Radhamani *et al.* observed that celastrol downregulateed phosphorylated STAT3 levels in a dose- and time-dependent manner, significantly inhibiting the translocation of STAT3 from the cytoplasm to the nucleus. Since STAT3 activation is mediated by soluble tyrosine kinases from the Src kinase family 109, this study further explored celastrol's effect on the constitutive activation of Src kinase in U266 cells, and reported that celastrol inhibited Src kinase phosphorylation over time 109. Given that the cytokine IL-6 is a primary stimulator and inflammatory mediator of STAT3, promoting tumor survival by blocking programmed cell death, treatment with celastrol was shown to inhibit IL-6-induced STAT3 phosphorylation in a time-dependent manner, reducing both constitutive and inducible STAT3 activation 20. Similarly, another study also showed that celastrol affects the IL-6/STAT3 signaling pathway in NSCLC cells. After treatment of H460, H520 and PC-9 cells with IL-6 and celastrol, IL-6 stimulation of STAT3 was reversed in a dose-dependent manner by celastrol. Immunofluorescence staining analysis also showed that IL-6 treatment induces cytoplasmic-nuclear P-STAT3 transfer, whereas celastrol restrained this transfer 41. Therefore, we hypothesize that celastrol may have potential for the treatment of various cancers through its interaction with STAT3.

### 3.3 Nur77 is a potential anti-tumor target of celastrol

Nuclear receptors (NRs) are a large superfamily of post-transcriptional factors involved in various biological processes, making them significant pharmacological targets. Various diseases, including diabetes, cardiovascular disease and cancer have been associated with compromised NR activity 110. Nur77, also known as NR4A1/TR3/NGFIB, features a typical nuclear receptor structure, and its expression and localization are closely related to its roles in apoptosis and cell proliferation. Although Nur77 is considered an orphan receptor owing to the unidentified nature of its endogenous ligand, an increasing number of small molecules that target Nur77, such as celastrol, have demonstrated therapeutic potential in cancer and other diseases 110.

Research indicates that celastrol binds to Nur77 via a specific non-covalent interaction, positioning celastrol near the sulfhydryl group (C551) of the active cysteine to form a reversible covalent bond 111. Further studies revealed that celastrol induces ubiquitination of Nur77's C-terminal LBD and its interaction with p62, resulting in the formation of Nur77-LBD/p62 condensates. This process also necessitated further interaction between Nur77's N-terminal IDR and p62 PB1, highlighting the importance of coordinated multivalent interactions between Nur77 and p62 112. Further investigations have shown that celastrol facilitates the transport of Nur77 from the nucleus to the mitochondria, where it engages with tumor necrosis factor receptor-associated factor 2 (TRAF2) to inhibit TNF-α-induced ubiquitination of TRAF2 and Lys63-linked ubiquitination of Nur77. This ubiquitinated Nur77 then interacts with p62/SQSTM1, inducing autophagy to remove damaged mitochondria. Additionally, the interaction of celastrol-induced Nur77 with TRAF2 obstructs the IKK-NF-κB pathway by curbing IKK-α/β phosphorylation and reducing the TNF-α-induced degradation of iκBα, thereby suppressing inflammation, and promoting autophagy 113. In addition, researchers have shown that Nur77 is one of the targets of celastrol and is associated with anti-colorectal cancer activity. Celastrol may upregulate Atg7 by targeting Nur77, resulting in increased autophagy, thus playing an anti-tumor role, which may provide a new understanding of the anti-tumor effect of celastrol in CRC 47. Overall, Nur77 is a vital target of celastrol, and more studies are needed to determine how celastrol targets Nur77 for its anti-tumor effects.

### 3.4 Celastrol and Nedd4

Nedd4 is an important E3 ubiquitin ligase protein, which is primarily involved in ubiquitination-dependent protein degradation in the lysosome, proteasome, and endoplasmic reticulum 114. Recent research has highlighted the critical role of Nedd4 in tumorigenesis by regulating various downstream signaling pathways. Salah *et al.* reported that Nedd4 directly interacts with and ubiquitinates LATS1, reducing LATS1 levels which inhibits the activity of the Hippo signaling pathway and suppresses tumorigenicity 115. Furthermore, Nedd4 is known to counteract the carcinogenic Notch signaling pathway by promoting the degradation of Notch and Deltex 116. Our recent study demonstrated that celastrol mitigated oxidative damage in cerebral ischemia-reperfusion injury (CIRI) by boosting the expression of nuclear factor E2-associated factor 2 (Nrf2). Proteomic methods showed that Nedd4 was the direct target of celastrol, and that significantly blocks ubiquitination of the Nedd4-K48 link and inhibits the degradation of Nrf2, thereby reducing the production of reactive oxygen species by astrocytes in CIRI and exerting a protective effect on neurons 117.

### 3.5 Celastrol and peroxiredoxin proteins

Peroxiredoxins (Prdxs) are a ubiquitous and highly conserved family of redox regulatory proteins capable of eliminating various reactive oxygen species (ROS). This family, consisting of mercaptan-dependent peroxidase enzymes, degrades hydrogen peroxide and plays a crucial role in maintaining and reacting to altered cellular redox homeostasis 11. The relationship between oxidative stress and cancer is complex, as normal cells rely on antioxidant systems to limit oxidant production, neutralize active substances, and repair oxidative damage. Disruption of these systems may lead to cancer 118.

Recent studies have demonstrated that celastrol targets Prdx proteins, which play critical roles in liver fibrosis and tumor management both *in vitro* and *in vivo* 38,98,119-120. A previous study suggested that celastrol might play an anti-inflammatory role by activating the AMPK-SIRT3 pathway to prevent liver fibrosis, yet the direct targets and precise mechanisms of celastrol remain unclear 121. Our research group developed an activity-based celastrol-probe (cel-p) to explore the direct targets of celastrol in liver fibrosis. We found that celastrol enhances ROS-mediated signaling pathways and induces ferroptosis in hepatic stellate cells (HSCs), effectively mitigating liver injury and fibrosis caused by carbon tetrachloride (CCl4). Through activity-based protein profiling (ABPP), we discovered that celastrol binds directly to Prdx1, Prdx2, Prdx4, and Prdx6 via active cysteine sites, inhibiting their antioxidant activity without affecting protein expression. Celastrol also targeted heme oxygenase-1 (HO-1), increasing its expression in activated hematopoietic stem cells. Knockdown of these target proteins in hematopoietic stem cells led to increased cell ROS levels and induced ferroptosis. Thus, our research identified specific protein targets and molecular mechanisms by which celastrol ameliorates liver fibrosis, laying a theoretical foundation for its potential as a therapeutic agent. Chen *et al.* combined biotin-labeled celastrol (bio-celastrol) with recombinant proteins on a HuProtTM human protein microarray to identify potential celastrol-binding proteins in gastric cancer cells. They reported that celastrol bound directly to Prdx2, inhibiting its enzyme activity, increasing cellular ROS levels and leading to ROS-dependent mitochondrial dysfunction, endoplasmic reticulum stress and apoptosis 38.

**Figure 4:**
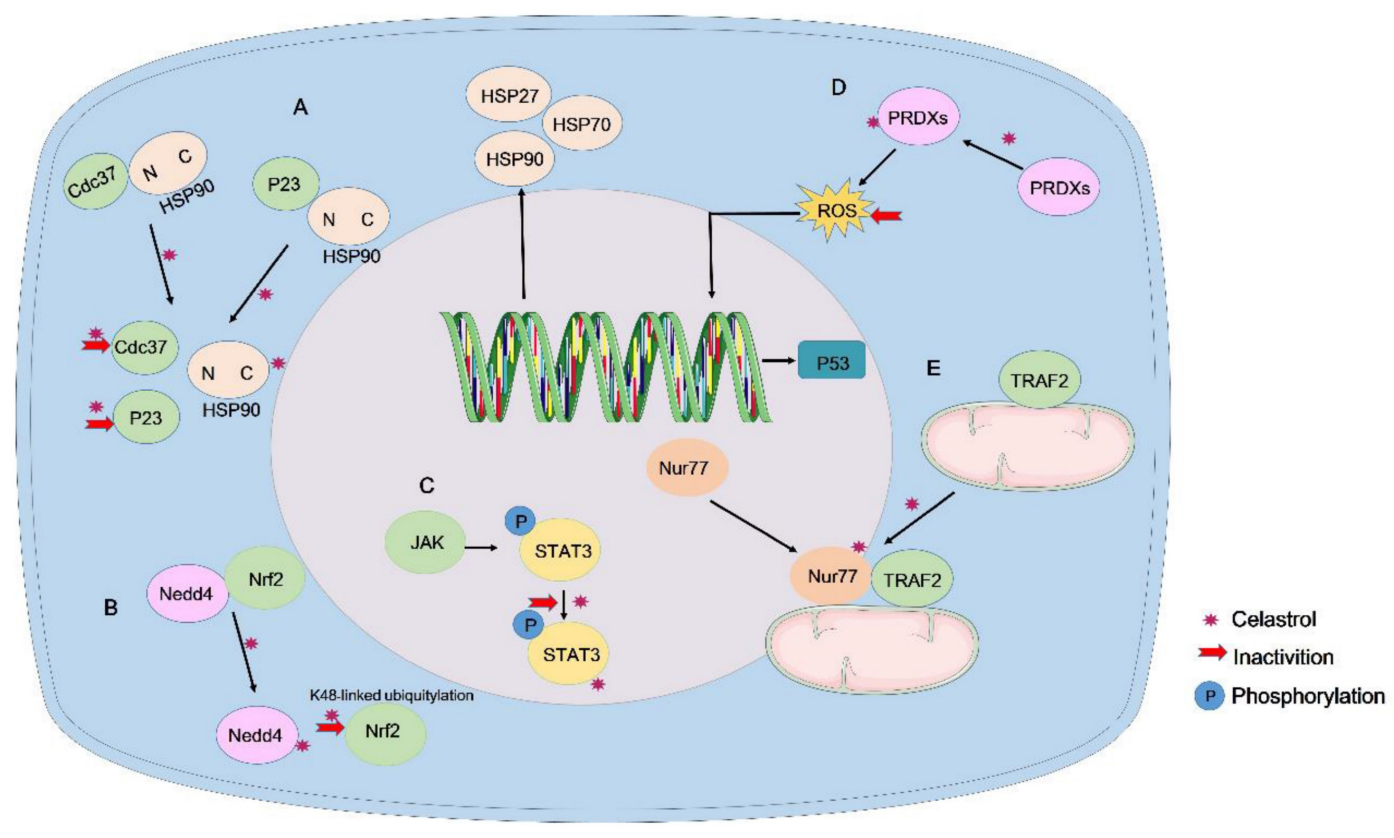
Direct binding targets of celastrol. (A) Celastrol covalently binds to the HSP90 co-chaperones Cdc37 and p23 to disrupt the Cdc37-HSP90 or p23-HSP90 complex. (B). Celastrol directly binds to Nedd4 and inhibits the interaction between Nedd4 and Nrf2, reducing the K48-linked ubiquitylation of Nrf2. (C). Celastrol directly binds and inhibits STAT3 tyrosine phosphorylation and nuclear translocation. (D). Celastrol targets Prdx1 to inhibit ROS production and suppress the proliferation of colorectal cancer cells. (E). Celastrol covalently binds to Nur77 and induces Nur77 interaction with TRAF2 to inhibit the classical IKK/NF-κB pathway.

Chen *et al.* developed a new computational tool, OTTER, and combined it with experimental analyses to determine that Prdx1 is a target protein of celastrol that regulates ROS in colorectal cancer. They resolved the high-resolution crystal structure of Prdx1 bound to celastrol and synthesized a novel derivative, 19-048, with strong anti-Prdx1 activity and selectivity towards Prdx2~Prdx6. Treatment with celastrol or its analogs upregulated the expression of cell cycle arrest and apoptosis-related genes in the p53 signaling pathway, effectively inhibiting the proliferation and inducing apoptosis in colorectal cancer cells 120. Consequently, the anti-tumor effect of celastrol may be attributed to the inhibition of peroxiredoxin activation. The multifaceted activity of celastrol could explain its therapeutic effects across various cancers (Figure 4).

Celastrol exerts its antitumor effects by binding to different targets. However, the synergism or antagonism between different targets has seldom been investigated. It was found that luteolin reduced the binding of HSP90 to STAT3 and released the phosphorylated STAT3 from the HSP90 chaperone proteasome, which may lead to the simultaneous exposure of phosphorylated STAT3 to phosphatases and proteases. This leads to a subsequent decrease in the level of STAT3 phosphorylation, thus inhibiting the viability of gastric cancer cells with inactivation of STAT3 and exerting anti-tumor effects 122-123. XL888, an HSP90 inhibitor, attenuates the formation of the HSP90 and STAT3 complex, leading to a decrease in the expression levels of STAT3 and p-STAT3. It also inhibits the expression levels of Mcl-1 and cleaved-caspase 3 *in vivo* and *in vitro*, and promotes heat-induced apoptosis in HCC cells 124. While there are currently no studies on the interaction between the targets of celastrol, we have hypothesized that celastrol may also play an anti-tumor role by reducing the binding of HSP90 to STAT3 and reducing STAT3 phosphorylation, and we will conduct experiments to verify our conjecture in the future.

## 4. Drug combination and bioavailability

### 4.1 Drug combinations

As an active component of Chinese medicine with promising anti-tumor prospects, the clinical application of celastrol is nevertheless limited by its severe hepatic and renal toxicity and low bioavailability 15. Currently, combination therapy has emerged as the preferred clinical treatment for various tumors 125,126, enhancing the efficacy of individual drugs and reducing their dosage and side effects, thereby improving patient tolerance. Additionally, the diverse mechanisms of action of different drugs minimize the risk of tumor cells developing resistance, making combination therapy an effective strategy to combat drug resistance 127. Hence, combining celastrol with other agents offers a novel avenue for cancer treatment.

The anti-tumor effects of celastrol are potentiated when used in conjunction with other therapeutic agents. Research has indicated that celastrol, combined with 17-*N*-Allylamino-17-demethoxygeldanamycin (17-AAG), inhibits the toxic stress response of HSP90-targeted proteins, reduces the sensitization of human glioblastomas to celastrol treatment, and increases the accumulation of polyubiquitylated aggregates and p62, thereby enhancing protein stability and overcoming multidrug resistance (MDR) in glioblastoma 128. Similarly, another study revealed that celastrol, when combined with EGFR tyrosine kinase inhibitors (EGFR-TKIs), effectively inhibits the growth and invasion of T790M mutant human lung cancer H1975 cells by suppressing the expression and phosphorylation of EGFR, STAT3, p-Akt, and p-ERK 129. These findings suggest that blocking the p-EGFR pathway could inhibit cancer cell invasion. *In vivo* experiments further confirmed that celastrol, when used with EGFR-TKIs, exhibited a significantly greater inhibitory effect on tumor growth than either agent alone. Moreover, when celastrol is used in combination with histone deacetylase inhibitors (HDACis), it upregulates H4K16 acetylation (H4K16ac), modulates H3K4 trimethylation and H3S10 phosphorylation, synergistically inhibiting cancer cell proliferation. Celastrol also significantly boosts the efficacy of HDACis in suppressing mouse allogeneic lung cancer cell grafts by upregulating H4K16ac 130. These findings suggest that the combination of celastrol and HDACis could represent a new therapeutic approach in cancer treatment. In summary, the results above hint to us that the synergistic sensitization of celastrol with other drugs may result in better anti-tumor outcomes.

### 4.2 Celastrol bioavailability

While celastrol demonstrates promising antitumor activity, its clinical application is hindered by several physicochemical and pharmacokinetic challenges, such as low water solubility, low bioavailability, and inadequate targeting 131. The advent of nanotechnology in drug delivery has introduced innovative approaches to cancer treatment. Nano formulations are characterized by their robust drug loading capacity, prolonged circulation *in vivo*, and the ability to target tumor tissues either passively or actively through control over particle size, structural modifications, and surface alterations 132. This emerging field offers potential solutions to enhance the therapeutic efficacy, oral bioavailability, and tissue targeting capabilities of celastrol 133.

Choi *et al.* formulated composite nanoparticles loaded with celastrol/aceitinib by loading celastrol into mesoporous silica nanoparticles and aceitinib into a pegylated lipid bilayer, which was approximately 120 nm in size and had a narrow polydispersity index (about 0.07). *In vitro* cytotoxicity experiments showed that the composite nanoparticles effectively inhibited angiogenesis and mitochondrial function, and effectively internalized rat squamous cell carcinoma SCC-7 cells, human breast cancer BT-474 cells and human neuroblastoma SH-SY5Y cells. Meanwhile, in the heterogeneous tumor model, the tumor inhibition rate of the composite nanoparticle group was significantly greater than that of the single agent group, reaching 64% 134. Liu *et al.* developed an innovative chemo-immune strategy based on targeted delivery of mitoxantrone and celastrol, and found that when mitoxantrone and celastrol were effectively co-delivered to the tumor site at the optimal ratio (5:1), immunogenic tumor cell apoptosis was significantly triggered and tumor antigen recognition was restored. This approach stimulated overall anti-tumor immunity, reducing drug dosage and adverse reactions through synergistic effects. The nano-vector-mediated chemo-immunotherapy successfully altered the fibrotic and immunosuppressed tumor microenvironment, impeding tumor growth and further inhibiting metastasis to major organs 135. Another investigation developed novel celastrol-loaded poly (ε-caprolactone) nanoparticles (cel-NPs) to assess their antitumor effects on prostate cancer cells. These cel-NPs dose-dependently inhibited cell proliferation across all the tested prostate cancer cell lines, particularly at low/medium doses (0.5 and 1.0 µM), and significantly enhanced the cytotoxicity against the DU145 and PC3 cell lines by modulating apoptosis and cell cycle regulatory proteins 136. Moreover, a self-assembled nanomedicine was made by coupling celastrol with p-selectin targeting peptide (PSN) and low molecular heparin (LMWH). The results of *in vivo* and *in vitro* experiments confirmed that the prepared nanomedicine enhanced the anti-tumor and anti-metastatic ability of celastrol and significantly reduced organ toxicity 137.

**Figure 5:**
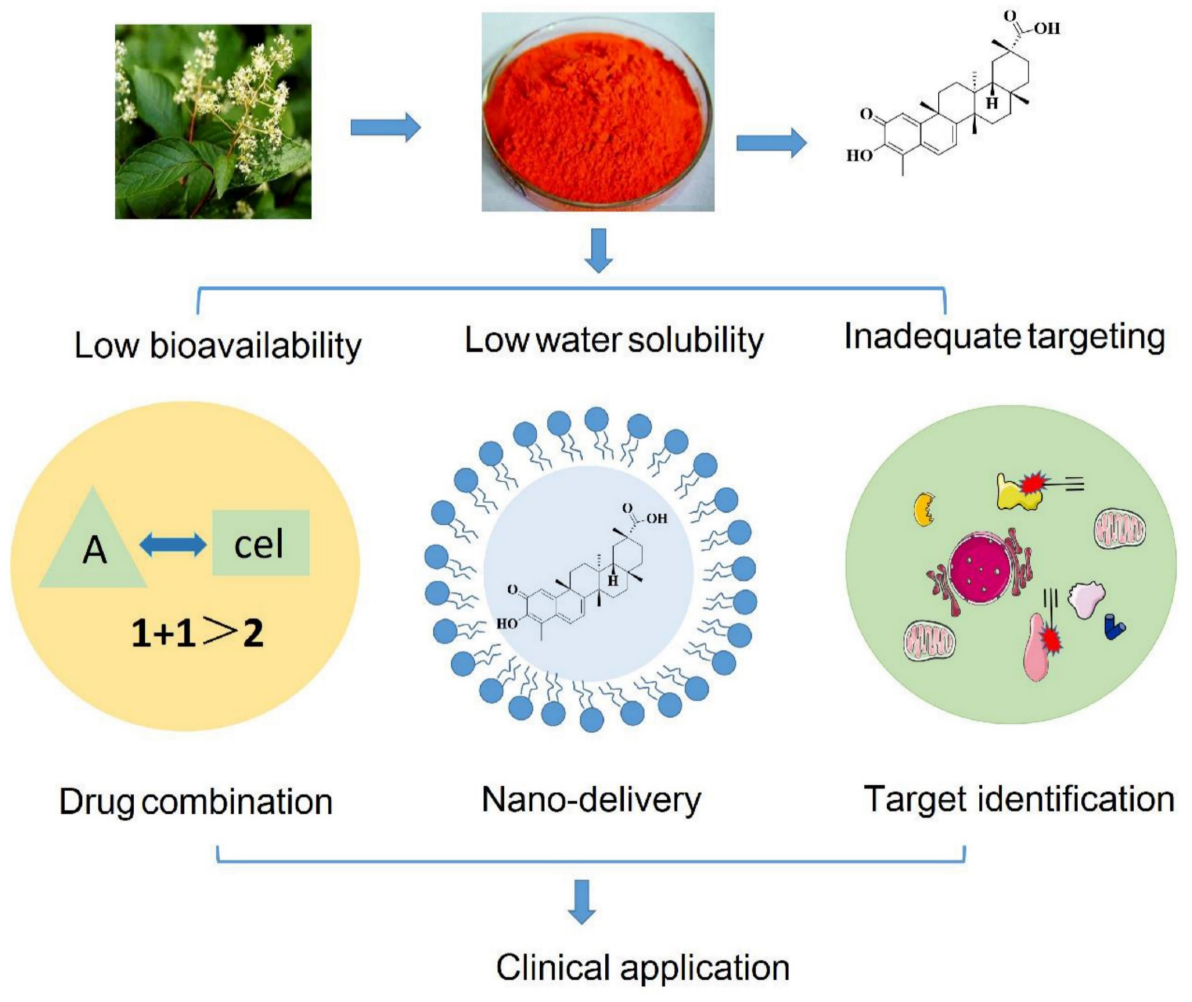
Strategies to improve the bioavailability of celastrol. We summarize the methods used to ameliorate the shortcomings of low bioavailability, low water solubility and inadequate targeting of celastrol, including drug combination, nano-delivery and target identification.

In summary, nanotechnology-based drug delivery systems offer a versatile platform for the simultaneous transport of one or more drugs, enhancing drug solubility, pharmacokinetic and pharmacological properties, and facilitating targeted drug delivery while minimizing accumulation in non-targeted organs. Therefore, nano-delivery systems present a novel pathway for the development and clinical application of celastrol, potentially overcoming existing limitations and maximizing its therapeutic potential. Strategies to improve the bioavailability of celastrol are shown in Figure 5 and Table 2.

## 5. Side effects of celastrol

**Figure 6:**
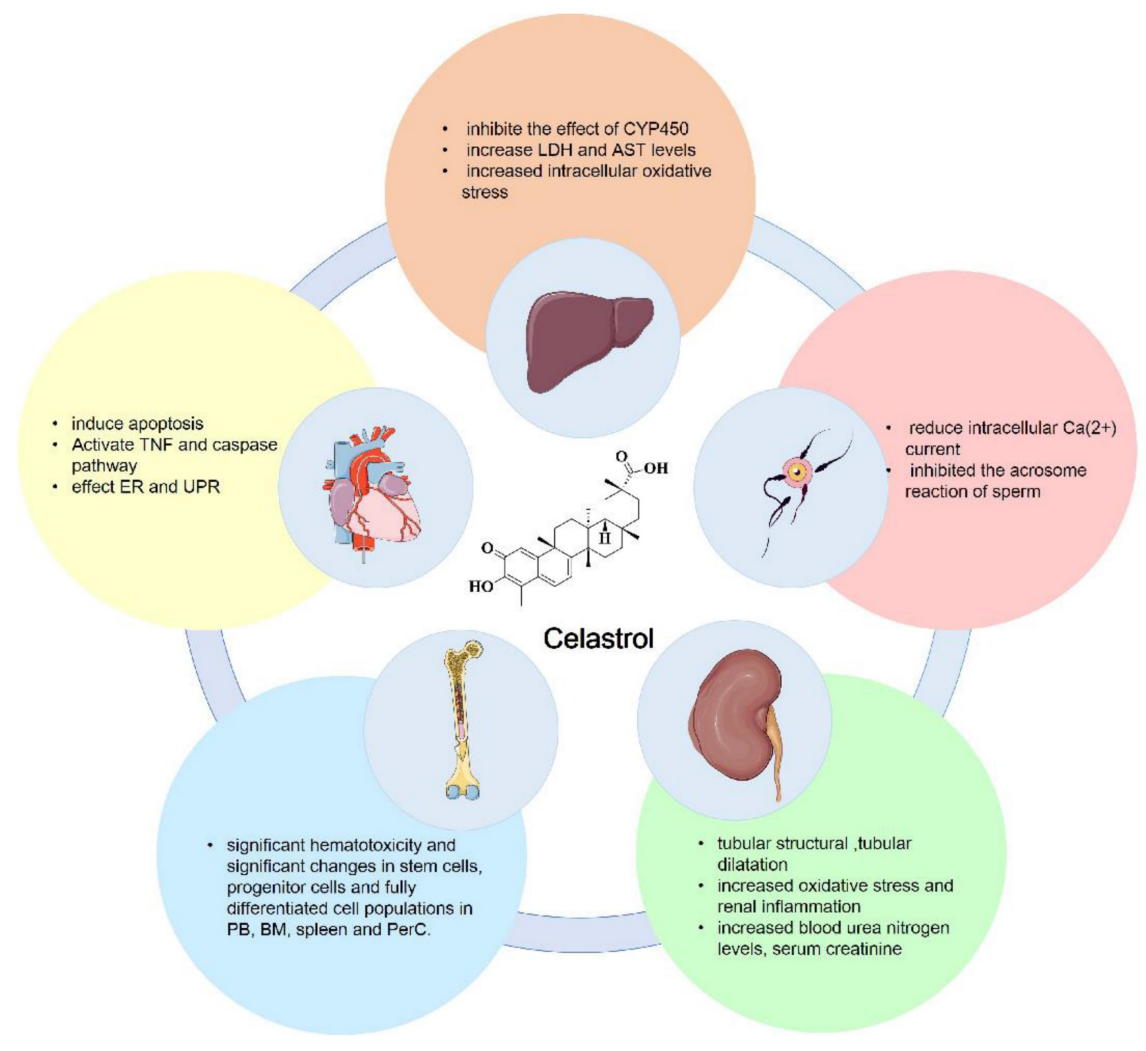
The main toxicities of celastrol include hepatotoxicity, cardiotoxicity, infertility toxicity, hematopoietic system toxicity and nephrotoxicity.

Currently, celastrol is mainly used in the clinical treatment of rheumatoid arthritis, ankylosing spondylitis, glomerulonephritis, lupus erythematosus and various skin diseases in the form of celastrol tablets, celastrol polyglycoside tablets, and celastrol extract tablets. Despite its therapeutic applications, the use of celastrol in cancer treatment is impeded by several significant challenges, including its pronounced hepatic and renal toxicity and potential for causing immunosuppression 15. Here, we summarize the toxicity of celastrol, which includes hepatotoxicity, cardiotoxicity, infertility toxicity, hematopoietic system toxicity and nephrotoxicity. The major toxicities of celastrol are shown in Figure 6.

### 5.1 Hepatotoxicity

Cytochrome P450 (CYP450) is part of a membrane-bound hemoprotein family that functions as a metabolic enzyme crucial for the detoxification, metabolism, and homeostasis of exogenous substances. Its induction or inhibition can alter metabolic pathways in the body and even affect the effectiveness and safety of drugs 138. The hepatotoxic effects of celastrol were examined by assessing the impact of CYP450 inhibition following 24 hours of administration *in vitro* 139. Treatment with celastrol substantially reduced the viability of rat primary hepatocytes, leading to increased levels of AST and LDH, as well as heightened intracellular oxidative stress. Phenobarbital (a CYP450 enzyme attractant) reduces the hepatotoxicity of celastrol, whereas 1-aminobentriazole (a broad-spectrum inhibitor of cyp450) potentiates the hepatotoxicity of celastrol 140.

### 5.2 Cardiotoxicity

Utilizing mass spectrometry-based metabolomics, network toxicology strategies and molecular biology, the mechanisms underlying celastrol-induced cardiac damage have been elucidated. The findings revealed that celastrol treatment significantly increased plasma palmitic acid levels in rats, leading to an oxidative stress response *in vivo*. This activation further stimulated the TNF signaling pathway and the caspase family, which induces apoptosis. Celastrol dose-dependently induced cardiac dysfunction in mice, manifesting as cardiomyocyte hypertrophy, left ventricular dilation and myocardial interstitial fibrosis, and significantly reduced the activity and promoted apoptosis of neonatal ventricular myocytes (NRVMs) in rats. More importantly, celastrol was found to exert its pro-apoptotic effect through ER stress and the unfolded protein response 141. Additionally, a study utilizing a zebrafish model to assess the toxicity of celastrol *in vivo* found that administering different concentrations of celastrol to embryos for 120 hours post-fertilization significantly reduced the incubation rate of embryos at concentrations of 1.0 μM or higher. This led to severe edema in the larval sac, tail bending or hook-like tail deformities, and pericardial edema 142. The above studies indicate that celastrol has obvious cardiotoxicity, suggesting that the cardiovascular effects of celastrol should be carefully evaluated during clinical use.

### 5.3 Infertility toxicity

Bai and colleagues investigated the impact of celastrol on Ca^2+^ channels and the acrosome reaction in mouse spermatogenic cells via whole-cell patch-clamping and chlortetracycline staining. Their findings demonstrated that celastrol significantly reduced intracellular Ca^2+^ currents in a time-dependent and irreversible manner, additionally inhibiting the progesterone-induced acrosome reaction in sperm. These observations suggest that celastrol may exhibit anti-fertility effects by impeding Ca^2+^ currents 143.

### 5.4 Hematopoietic system toxicity

Researchers have explored the hematotoxic effects of celastrol by administering 5 mg/kg celastrol intraperitoneally to 8-10-week-old BALB/c mice daily. After four days, the mice in the administered group developed a hunched posture and wrinkled fur compared with those in the control group, and cells from the peripheral blood (PB), bone marrow (BM), spleen, and peritoneal cells (PerC) of mice were collected and analyzed. The results showed that celastrol treatment led to significant hematotoxicity and marked alterations in stem, progenitor, and fully differentiated cell populations in the PB, BM, spleen, and PerC of mice. These results underscore the importance of understanding the toxicity and side effects of celastrol to better inform its clinical use 144.

### 5.5 Nephrotoxicity

Some research has demonstrated that treatment with celastrol exacerbates LPS-induced acute kidney injury in mice, which is mainly characterized by damage to renal tubular structure (loss of brush border) and tubular dilatation, increased oxidative stress and renal inflammation, as well as elevated blood urea nitrogen, serum creatinine, neutrophil gelatinase-associated lipocalcin, and kidney injury molecule-1 levels 145. These findings highlight the nephrotoxic potential of celastrol, urging clinicians to carefully consider its nephrotoxic effects when it is prescribed. Despite the known toxic properties of celastrol, comprehensive data are still scarce. Consequently, further research is necessary to elucidate the mechanism of action of celastrol and its role in various disease models, aiming to gather more detailed and reliable data for a better understanding and application of celastrol in clinical settings.

## 6. Anti-inflammatory effects of celastrol in other diseases

Celastrol has anti-inflammatory and immunosuppressive effects that can be used to treat a variety of inflammatory and immune disorders, such as asthma, rheumatoid arthritis, inflammatory bowel disease and systemic lupus erythematosus. Studies have reported that celastrol inhibits TNF-α-induced proliferation of fibroblast-like synoviocytes (FLSs) decreases the secretion of pro-inflammatory cytokines while increasing the levels of autophagosomes and expression of LC3-II and Beclin-1 proteins in TNF-α-treated FLSs, and decreases the phosphorylation of mTOR and AKT thereby reducing the degree of rheumatoid arthritis 146. In addition, celastrol inhibits the repolarization of macrophages to the pro-inflammatory M1 phenotype by modulating the NF-κB and Notch1 pathways, which significantly reduces the secretion of a variety of pro-inflammatory cytokines and inhibits the progression of RA 147. Celastrol covalently binds to and dissociates the COMMD3/8 complex, a signaling adapter for chemoreceptors, inhibiting B-cell migration, decreasing antibody responses, and halting the progression of RA 148. Based on the integration of systems pharmacology, proteomics, transcriptomics, and single-cell transcriptomics, it has been revealed that celastrol may exert immunomodulatory effects and thus treat RA by modulating the PI3K/AKT signaling pathway through the regulation of key targets such as TNF and IL6 149. Celastrol can also play a therapeutic role in a variety of inflammatory and autoimmune diseases by regulating various signaling pathways and inducing different responses in targets such as nuclear factor kappa B (NF-κB), mitogen-activated protein kinase (MAPK), PI3K Akt-Mtor, Ednrb/Kng1, NLRP3, AMPK-SIRT3, and Ca^2+^ 150.

## 7. Discussion

Compared with synthetic compounds, natural products constitute an important part of the pharmaceutical market because of their safety, efficacy, and low cost 7. Celastrol, a standout natural compound, has emerged as one of the five most promising natural drugs due to its reported effectiveness against a broad spectrum of tumors, including those of the breast, colon, lung, adipose, liver, and pancreas, both *in vivo* and *in vitro*. In this review, we conclude that the effective amelioration and treatment of multiple tumors by celastrol is closely related to a variety of molecular mechanisms, such as the inhibition of cell proliferation, the regulation of cell cycle, apoptosis and autophagy, the inhibition of cell invasion and metastasis, and anti-inflammatory, regulation of immunotherapy and angiogenic mechanisms. However, the current research on the anti-tumor effects of celastrol remains lacking, and further in-depth studies are needed in the future to reveal the MOA of celastrol in tumor treatment.

Currently, celastrol is widely used to treat rheumatoid arthritis (RA), ankylosing spondylitis (AS), systemic lupus erythematosus (SLE) and Crohn's disease (CD). Meanwhile, celastrol has also been reported to have great potential for the treatment of tumors, but its poor water solubility, high toxicity, low bioavailability, and rapid metabolism limit its clinical application. In this review, we have summarized the side effects of celastrol, as well as methods to ameliorate these side effects and low bioavailability of celastrol, including combination drugs and nano-administration. In addition, in order to reduce the toxicity of celastrol and improve its bioavailability, Tan and colleagues prepared a mitochondria-targeting nanoparticle by modifying liposome carrier CSOSA with lipophilic cationic CTPP. It has been demonstrated that micelles enable rapid release of celastrol into mitochondria, reduce drug leakage into the cytoplasm and lysosomes, and improving the anti-tumor capacity of celastrol 151. Additionally, researchers have summarized the major functional groups on celastrol that can be modified, including the C-2/3 oxygen, C-6 hydrogen, C-20 carboxyl, and quinone-methyl portions of the A- and B-rings, as well as their structure-activity relationships, which provide guidance for the development of celastrol analogs or derivatives 152. In addition, a variety of delivery methods can be used to increase the bioavailability and reduce the toxicity of celastrol. These include glucolipid-celastrol conjugates, nucleic acid aptamer-celastrol conjugates, dendrimers, albumin, polymers, and vesicular carriers 153. Therefore, celastrol can be used for clinical antitumor applications based on its optimized structural design, reduced toxicity, and improved bioavailability.

## 8. Conclusion

Alongside improvements in quality of life and increasing lifespans, the incidence of cancer has also increased in modern times, posing an enormous burden in terms of health and economic costs. Despite the proliferation of therapeutic approaches and targeted drugs, cancer remains the leading cause of death worldwide. Therefore, there is an urgent need for further research into new strategies for cancer treatment and the discovery of potential new drugs. This review comprehensively describes the current mechanisms of action and targets of celastrol in cancer, providing promising preclinical evidence for its treatment of cancer. Despite the positive therapeutic potential of celastrol, concerns about its clinical development remain due to its poor pharmacokinetic profile, potential toxicity and lack of long-term data. With the availability of various nano-formulations, combination therapies, analogs and other therapies, the development of celastrol formulations seems promising. However, more information on its dosage, formulation, efficacy, and toxicological profile needs to be obtained before the drug can enter human trials. In conclusion, the present review comprehensively reviews and summarizes the molecular mechanisms and targets of celastrol, providing new research ideas for improving therapeutic strategies, reducing side effects, expanding new fields and establishing a theoretical basis for the further development and clinical application of celastrol.

## Figures and Tables

**Table 1:** Summary of detailed experimental study of celastrol against different tumor types in this article.

Effects of celastrol on cancers	Cancer Type	Cell line	Dose of administration	Mechanisms of action of celatrol	Ref.
Inhibits cell proliferation and regulates cell cycle	ovarian cancer	A2780, SKOV3, OVCAR3	0, 0.25, 0.5, 1, 2 and 4 μM	inhibited Pin1 protein, CyclinD1↓, CDK2↓, CDK4 ↓genes	18
		A2780 and SKOV3	0, 0.1, 0.3, 1, 3, and 10 µM	induced cell cycle arrest in G2/M phase with Cyclin B1↑ and Cyclin E↓	19
	Multiple myeloma	U266, RPMI 8226, RPMI-8226Dox-6, RPMI-8226-LR-5	0, 0.1, 0.25, 0.5, 1, 2.5 and 5 µM	inhibited cell proliferation, ub-G1 phase arrests cyclin D1↓ and cyclin E↓, p21↑ and p27↑	20
	colon cancer	LOVO, LOVO/DX	0, 0.1, 0.5, 1.5, 10 and 20 µM	increased ROS amount, arrest in the S and G2/M phase	21
	osteosarcoma	HOS, MG-63, U-2OS, Saos-2	0, 1, 2, 3, 4 µM	induced apoptosis via activation of the OSs/JNK signaling pathway, cell cycle arrest at the G2/M phase	22
	gastric cancer	BGC-823, MGC-803	2 μM	inhibited miR-21-mTOR signaling pathway, p27↑, induced G2/M cell-cycle arrest in gastric cancer cells	23
	nasopharyngeal carcinoma cancer	NPC-039, NPC-BM, Cis-039, Cis-BM	0, 1, 2, 4 µM	induced cell cycle arrest in the 2G /M phase of nasopharyngeal carcinoma by up-regulating p38 MAPK and ERK pathway, and induces apoptosis mediated by endogenous and exogenous apoptotic pathways, thus playing an anti-tumor role	24
Regulates cell apoptosis	hepatocellular carcinoma	HepG2	10, 20 and 40 μM	inhibited the phosphorylation of mTOR, cleaved-PARP↑, cleave-caspase-3↑, cleave-caspase-8↑, cleave-caspase-9↑	26
	Canine mammary tumor	CMT-7364	0, 0.1, 0.4, 0.6, 0.8, 1.2 and 1.6 µM	inhibited the proliferation, reduced the migration and invasion, cleaved caspase-3 ↑ and cleaved caspase-9 ↑, induced cell apoptosis	27
	ovarian cancer	OVCAR3	0, 0.25, 0.5, 1, 2, 4, 6 µM	inhibited PI3K/ Akt-NF-κB signaling pathway, induced apoptosis and inhibited cell proliferation caspase-3↑ and caspase-9↑	28
	non-small-cell lung cancer	A549	0, 0.5, 1, 2, 4, 8 µM	activated mitochondria and Fas/Fas related pathway, Bax↑, Bcl-2↓, inhibited Akt phosphorylation	32
	glioblastoma	U87-MG, U251, and LN229	0, 0.5, 1, 2, 4 µM	activated cell death receptor pathway, DR5↑, caspase-8↑, caspase-3↑and PARP↑, inhibited tumor cell proliferation and inducing cell apoptosis	33
	Nasopharyngeal carcinoma	HONE-1 and NPC-039	0, 1, 2, 4 µM	TNRSF1A↑ and 10B↑, TNFRSF1A associated via death domain↑, Fas↑, Fas-associated via death domain↑, lead to cell cycle G1 phase and G2/M phase and induced cell apoptosis	34
	Acute leukemia	HL-60 cells and NB-4	0, 0.125, 0.25, 0.5 µM	induced cell apoptosis by inhibiting cysteine metabolism, increased reactive oxygen species (ROS) level, and activated mitochondrial apoptosis pathway	35
	gastric cancer	SGC-7901 and BGC-823	0, 1, 2, 3 µM	inhibited the activity of Prdx2 to increase ROS levels, leading to ROS-dependent ER stress, mitochondrial dysfunction and apoptosis	38
	osteosarcoma	MG- 63, U-2OS and HOS	0, 0.5, 1, 2, 4, 6 µM	triggered mitochondrial apoptosis pathway, Bax↑, cytochrome c↑, procaspase-9↓, cleavaged PARP↓	36
	Hepatocellular carcinoma	HepG2 and Bel7402	0, 0.625, 1.25, 2.5, 5.0 and 10 μM	inhibited the levels of ERS-related proteins, mitochondrial apoptosis-related proteins and autophagy related proteins, cause G2/M phase rest and inhibit proliferation	39
	Cervical cancer	HeLa	0, 0.5, 1, 2, 4 µM	up-regulated the expression of Bip, CHOP, XBP1s and IRE1 proteins, increase the transcription of endoplasmic reticulum stress target genes such as BIM, induced Bax translocation into mitochondria, and finally activated the endoplasmic reticulum and mitochondrial apoptosis pathway	40
	Non-small cell lung cancer	H460, PC-9, H520 and BEAS-2B	0, 1, 2, 4 µM	increased intracellular ROS levels, activated ERS pathway and inhibited P-STAT3 pathway, induced cell apoptosis	41
Regulates cell autophagy	prostate cancer	LNCaP, 22Rv1, DU145 and PC-3	2 µM	suppressed cell viability and upregulated autophagic activity, Atg5↑ and Atg7↑, increased autophagosome formation and promoted p62 degradation	46
	colorectal cancer	NCM460, HCT-116, SW480	0, 1.25, 2.5, 5 µM	induced apoptosis and autophagy by inhibiting Nur77, induced high expression of Atg 7 and Nur77/ Atg 7 signaling pathways	47
	cervix, lung and prostate cancer	HeLa cells, A549 cells and PC-3	0.5, 1.2, 4 µM	triggered autophagy and lipid droplet degradation, activated LXRα signaling	49
	gastric cancer	MKN1, MKN45, SNU216, SNU-668, and YCC-2	0, 0.25, 0.5, 1 µM	enhancement of autophagy-related proteins Atg 5↑, Atg 7↑, LC3 I↑, LC3 II and Beclin1↑	50
Inhibits cell invasion and metastasis	Hepatocellular Carcinoma	HCC, HepG2 and Hepa3B	0, 0.1, 0.3, 0.625, 1 µM	attenuated the proliferation and migration capacity of cells, CXCR4↓, PI3K, Akt	54
	colon and pancreatic cancer	MCF-7, KBM-5, SCC-4, Caco-2, HCT116, MDA-MB-231	0, 1, 2, 3 µM	regulated the expression of CXCR4, thereby inhibiting the invasion of tumor cells	55
	cervical cancer	HeLa	1, 10, 100 μM	inhibited the proliferation, invasion and migration of HeLa, MMP- 2↓ and MMP- 9↓.	58
	colorectal cancer	CRC cells SW480 and HCT116	0, 0.1, 0.5, 1, 2 µM	affected the PI3K/AKT signaling pathway, inhibited the proliferation and migration of colorectal cancer cells, MMP3↓ and MMP7↓	61
	osteosarcoma	U-2OS	0, 2.5, 4 µM	suppress the cell invasion and migration, inhibited the PI3K/Akt/NF-κB signaling pathway, MMP-2↓and MMP -9↓	60
	Malignant cancer, lung cancer	B16F10 and 95-D	0, 1, 2, 4, 8 µM	down-regulated the phosphorylation of FAK, activated the p38 MAPK signaling pathway, inhibited the adhesion plaque dependent cell migration and invasion	62
	Colorectal cancer	HCT116 and SW480	0.75 µM	reversed macrophage polarization from M2 to M1 by affecting the colorectal tumor microenvironment, polarized the macrophages through the MAPK signaling pathway	64
	Retinoblastoma cancer	Y79 cells	0, 0.5, 1, 2, 4 µg/mL	inhibited the HIF-1α /VEGF/VEGFR signaling pathway, ameliorated the hypoxic microenvironment of the tumors, and hindered the proliferation, migration, and invasion of vascular endothelial cells	65
Cel regulates tumor immunotherapy	Melanoma	B16F10, A375 and M10 cell	0, 0.1, 0.2, 0.5,1, 2, 4, 8 µM	induced immunogenic cell death, down-regulated PD-L1 expression, activated both dendritic cells and T cells, interrupted the PD-1/PD-L1 pathway	66
	Melanoma	B16F10	0.5 μg/mL	induced ER stress, promoted the recruitment and maturation of dendritic cells, leading to an increase in the proliferation of cytotoxic T lymphocytes (CD8+ T cells) and ultimately improving the efficacy of immunotherapy against melanoma	67
	melanoma	B16F10	0, 1, 2, 4,6, 8, 10 µM	bond to IL-2, disrupted the binding of IL-2 and CD25, markedly inhibited the proliferation and signaling of IL-2-dependent mouse T cells, and increased the number of CD8+ T cells, thereby inhibiting tumor growth	68
Anti-inflammatory properties of celastrol	Multiple Myeloma	U266, H929, and KMS11	0, 1, 2.5, 5 µM	inhibited the activation of NF-κB, CXCR4↓, MMP-9↓, reduced serum IL-6 and TNF-α levels	75
	ovarian cancer	OVCAR-3 and SKOV-3	0, 0.125, 0.25, 0.5, 1. 2 µM	blocked the NF-κB pathway, inhibited i-κBα phosphorylation, prevented i-κBα degradation and NF-κB accumulation, MMP-9↓, exerted anti-invasion activity of cancer cells	76
	Breast Cancer	MDA-MB-468 and MDA-MB-231	0, 0.1, 0.5, 1, 2, 5 µM	repressed migration and invasion through decreasing IL-6 levels, inactivated NF-kB signaling	77
	Hepatocellular Carcinoma	C3A, HepG2, Hep3B, and PLC/ PRF5	0, 0.5, 1, 2.5, 5 µM	inhibited STAT3 activation, inhibited the activation of kinases c-Src, and janus activates kinases -1 and -2 (JNK 1/2), affected tumor cell proliferation	79
	lung adenocarcinoma	A549	0, 0.5, 3, 4.5, 6 µM	up-regulated the expression levels of miR-24 and miR-181b, decreased STAT3 phosphorylation and inactivation and Bcl-2/ Bax ratio, inhibited cell proliferation and induced cell apoptosis	80
celastrol and angiogenesis	glioma	SHG44	0, 1, 2, 4 mg/kg/ day;	inhibited the growth of human glioma xenografts in nude mice, inhibited the expression and transcription of VEGFR1 and VEGFR2	83
	colorectal cancer	HT29, and HCT116	0, 2.5, 5, 10 µM	inhibited the growth and migration of cells inhibited the expression of key genes and proteins associated with the angiogenesis pathway	84
	prostate cancer	HUVECs or PC-3 cells	0,0.1, 0.5, 1, 2 µM	inhibited tumor angiogenesis and tumor growth *in vitro* and *in vivo*, targeted the AKT/mTOR/S6K kinase signaling pathway	14

**Table 2:** Strategies to improve the bioavailability and reduce the toxicity of celastrol.

Strategy	Drugs/ Drug delivery	Cancer Types	Concentration/IC_50_ *In Vitro*/Drug Dose *in Vivo*	Mechanism of Action	Aim	Ref.
Drug combination	Celastrol and 17-AAG /direct	Glioblastoma	celastrol alone LD50: 1.03±0.12 *μ*M without 17-AAG versus 0.69±0.11 *μ*M with 17-AAG	increased the accumulation of polyubiquitylated aggregates and p62, thereby enhancing protein stability and overcoming multidrug resistance (MDR) in glioblastoma	enhanced the antitumor efficacy and reduce the toxicity	128
	Celastrol and HDACi/direct	Lung cancer	Erlotinib (100 mg/kg/day/mouse), celastrol (1 mg/kg/day/ mouse), or erlotinib + celastrol	suppressed the expression and phosphorylation of EGFR, STAT3, p-Akt, and p-ERK	enhanced the antitumor effects of EGFR-TKI on NSCLC.	129
	Celastrol and HDACi/direct	Lung cancer	MS275 (5 mg/kg), celastrol (3 mg/kg), celastrol plus MS275 (3 mg/kg + 5 mg/kg, for 4 weeks	celastrol significantly boosts the efficacy of HDACi in suppressing mouse allogeneic lung cancer cell grafts by upregulating H4K16ac	enhanced the suppressive effects of HDACi on lung cancer	130
Nano formulations	Celastrol and aceitinib/ nanoparticles	multi-targeted cancer	Injected with 1 mg/kg of aceitinib, celastrol, aceitinib+celastrol cocktail	inhibited angiogenesis and mitochondrial function,	the tumor inhibition rate of the composite nanoparticle group was significantly higher than that of the single agent, reaching 64%.	134
	Celastrol and mitoxantrone/ nanocarrier	Desmoplastic Melanoma	celastrol alone IC50: 2.5 *μ*M; mitoxantrone alone IC50:16 *μ*M; celastrol + mitoxantrone (5:1) IC50:0.9/4.5 *μ*M	triggered apoptosis of immunogenic tumor cells and restoration of tumor antigen recognition	reduced drug dosage and adverse reactions	135
	celastrol/ nanoparticles	prostate cancer	celastrol IC50: <2.5 *μ*M; cel-NPs IC50: 0.5 or 1 *μ*M	modulated apoptosis and cell cycle regulatory proteins	enhanced the cytotoxicity against DU145 and PC3 cell lines	136
	celastrol/ nanodrug	primary and metastatic cancer	celastrol formulations (Cel, LC-NP, and PLC-NP) at a dose of 2 mg/kg every day for a total of 5 times.	inhibited PI3K/Akt/mTOR signaling pathway and ROS-mediated mitochondrial dysfunction	enhanced the anti-tumor and anti-metastatic ability of and reduced organ toxicity	137

## Abbreviations

TNRSF: TNF receptor superfamily members
CytC: cytochrome c
ROS: reactive oxygen species
MOA: mechanism of action
Prdx2: peroxoredotoxin-2
ER: Endoplasmic reticulum
NSCLC: non-small cell lung cancer
LC3I: Microtubule-associated protein 1 light chain 3I
CXCR: Chemokine receptors
NF-κB: nuclear factor-κB
STAT3: signal transducers and transcriptional activator 3
JNK 1/2: Janus activated kinases -1 and -2
Bcl-2: B cell proportional lymphomato-2
Bax: Bcl-2 associated X
VEGF: vascular endothelial growth factor
HIF-1: hypoxia-inducing factor
VEGFR: Vascular Endothelial Growth Factor Receptor
VDGFR: vascular dermal growth factor receptor
MAPK: Mitogen-activated protein kinase
ERK: Extraneous signal-regulated Kinase
EB: Eupalinolide B
SPR: surface plasmon resonance
CIRI: cerebral ischemia-reperfusion injury
Nrf2: nuclear factor E2-associated factor 2
Prdxs: Peroxredoxins
ABPP: Activity-Based Protein Profiling
HO-1: heme oxygenase-1
FLSs: fibroblast-like synoviocytes
